# Contemporary profiles and professional activities of French chiropractors: a national survey

**DOI:** 10.1186/s12998-025-00602-2

**Published:** 2025-10-13

**Authors:** Anne-Laure Meyer, Mathieu Picchiottino, Arnaud Lardon, André Bussières

**Affiliations:** 1 Institut Franco-Européen de Chiropraxie (IFEC), Ivry-Sur-Seine, France; 2 Société Franco-Européenne de Chiropraxie (SoFEC), Lille, France; 3 Département Chiropratique, Université du Québec à Trois-Rivières, Trois-Rivières, QC Canada; 4 Faculty of Medicine, School Physical and Occupational Therapy, McGill University, Montreal, QC Canada

**Keywords:** Chiropractic, Survey, Questionnaire, Practice patterns, Interprofessional relations, Referral and consultation, France

## Abstract

**Background:**

A comprehensive description of the profile of chiropractic practices and services provided can help guide curriculum design, inform quality improvement and guideline initiatives, and facilitate workforce planning. This cross-sectional study aimed to describe the sociodemographic characteristics, professional activities, work organization, clinical practice patterns, and interprofessional referrals of chiropractors in France.

**Methods:**

A 37-item online questionnaire was administered between February and April 2023 to all registered chiropractors practicing or having a chiropractic-related professional activity in France, for whom an email address was available (n = 1067). We computed means and standard deviations for continuous variables and percentages for categorical variables. Representativeness of the results was estimated by comparing survey participants’ demographic information with members of the *Association Française de Chiropraxie* (AFC).

**Results:**

The response rate was 46.4% (67.7% females; mean age = 34.9 ± 9.7 years), a sample comparable to AFC members. Most participants graduated in the past 15 years (81%) from the *Institut Franco-Européen de Chiropraxie* (IFEC) (94.5%). Just over one fifth of respondents had an additional university degree, and a similar proportion were involved in at least one chiropractic-related professional activity, mainly as lecturers in the IFEC or supervising clinicians in its outpatient clinics. Over half of the respondents (53.5%) had their main practice location in one of the four most populated regions in France, with 27.4% working in a multidisciplinary setting. Chiropractors reported regularly referring patients to general practitioners and physiotherapists, but less commonly received referrals. Middle-aged adults most commonly sought care, and spinal pain was the primary complaint reported by chiropractors. Chiropractors generally provided advice and education, manual therapy, and exercises. Two-thirds of respondents reported feeling moderate (30.6%) to intense or very intense (31.7%) competition with other manual therapy practitioners.

**Conclusion:**

The French chiropractic workforce appears predominantly composed of female practitioners who graduated in the past 15 years. Practice patterns and continuing education choices suggest that evidence-based information is part of their practice. While chiropractors commonly reported referring patients to other care providers, fewer reported receiving referrals. Patients seeking chiropractic care were similar to those described in other countries.

**Supplementary Information:**

The online version contains supplementary material available at 10.1186/s12998-025-00602-2.

## Introduction

Musculoskeletal disorders (MSDs) represent a major public health issue worldwide, with low back pain remaining the leading cause of disability since the 1990s, contributing more years lived with disability than other non-musculoskeletal chronic conditions, such as chronic respiratory diseases, mood disorders, and diabetes [1]. In high-income countries, including France, the increasing prevalence of MSDs, partly driven by an aging population, raises concerns about access to equitable, high-value rehabilitation care [1–4]. Given that international clinical practice guidelines recommend non-pharmacological care for most patients with MSDs [5–8], chiropractors appear well positioned to help meet the growing needs of this population, considering their training and scope of practice [9–11].

In France, chiropractors are recognized as first-contact providers within the primary healthcare system. They have the legal authority to diagnose and manage patients with MSDs using non-surgical, non-pharmaceutical therapeutic modalities, including patient education, exercise, and manual therapy. Additionally, they are expected to refer more complex cases to medical specialists when deemed necessary, including radiologists [11]. French chiropractors have recently been authorized to perform musculoskeletal ultrasound [12], while other medical imaging must be performed by a medical specialist. Like mainstream medicine, French chiropractors are expected to follow a patient-centered biopsychosocial approach within an evidence-based framework [11, 12].

Despite the increasing demand for MSD-related rehabilitation care [3, 13], chiropractic in France faces challenges related to its integration into the national healthcare system. Unlike other countries, such as Switzerland and Denmark, where chiropractic is fully recognized as a medical discipline and seamlessly integrated into healthcare services [14, 15], chiropractic care in France remains outside the national health insurance system. Although some private insurers offer partial reimbursement, patients generally bear most of the cost of chiropractic services. Both the regulatory framework for the chiropractic profession and the scope of practice have evolved favorably, albeit slowly. While the first chiropractor began practicing in France in 1920 [16], it was not until 1984 that the *Institut Franco-Européen de Chiropraxie* (IFEC) established the country's first chiropractic training program. Legal recognition followed in 2002 [17], with subsequent decrees in 2011 and 2018 further clarifying professional practice [11] and educational requirements [12]. Today, practicing chiropractors in France must register with Regional Health Agencies as healthcare professionals.

A recent French government-commissioned report in France highlighted the need for high-quality data on chiropractic practice to support its integration into patient care pathways [18]. Yet, little information is available regarding the French chiropractic workforce, including their sociodemographic characteristics, professional activities, work organization, clinical practice patterns, and interprofessional referrals. This study aims to address this knowledge gap by providing the first comprehensive job analysis of the chiropractic profession in France.

## Methods

The study ethic approval for this cross-*s*ectional survey was granted by a local ethics review board at IFEC (IRB # 22_07_001).

### Study population and recruitment

To be eligible to participate, chiropractors had to be registered with at least one French Regional Health Agency and in practice—for at least one of their practice locations (when several)—in France, and/or have had at least one professional activity related to chiropractic in France. Retired chiropractors, those practicing exclusively in another country or without a professional activity related to chiropractic in France were not eligible.

Currently, no comprehensive registry of French chiropractors exists, and their exact number remains uncertain [18]. In 2022, estimates ranged from 1364, based on the mandatory pension fund for self-employed chiropractors in France, to approximately 1400, based on the *Association Française de Chiropraxie* (AFC), which is the main professional association with 1032 members that year (personal communication, 31 March 2022). Notably, no French administration body maintains or discloses a reliable list of French chiropractors, and membership in a professional association is not mandatory. To compile a sampling frame, all French chiropractic associations (n = 6) and the IFEC were contacted and asked to provide a list of their members. Three associations did not respond, and one other declined to participate. In total, 1067 French chiropractors were invited to participate in the survey using membership lists from the two main French chiropractic associations, i.e., the AFC and the *Association Française de Chiropraxie Pédiatrique* (AFCP), and the list of graduates from IFEC. Duplicate entries across sources were removed before sending invitations.

### Survey instrument

The survey questionnaire was developed based on similar surveys conducted in other countries, such as the 2009 Swiss [19], 2014 Danish [20], and 2017 Australian [21] job analyses. The items for the present job analysis were selected to fit with the French context, striking a balance between comprehensiveness and the number of items that would be acceptable for participants. As it was developed specifically for the purposes of this survey, the questionnaire had not been previously validated. The final survey encompassed 37 items designed to collect (i) sociodemographic information, (ii) professional activities and work organization, (iii) clinical practices, including patient characteristics and management, and (iv) interprofessional referrals (Additional file 1). There were 15 multiple-choice questions, 11 single-choice questions, and 11 open-ended questions. Chiropractors involved in professional activities (e.g., teaching at IFEC) but no longer in clinical practice were only asked to complete items 1–8.

The survey was reviewed for face validity by three licensed chiropractors who are leaders of the national chiropractic associations, and a senior researcher, all with in-depth knowledge of the chiropractic profession in France. Two of the association representatives are women with over 10 years of clinical experience as practicing chiropractors, being in practice at the time of the survey. The third had practiced as a chiropractor for more than 15 years before taking on leadership responsibilities, while also being a lecturer at IFEC. The senior researcher, also trained as a chiropractor but no longer in practice, brought expertise in several areas, including on the conduct of surveys. They were contacted by email to provide feedback on (i) the alignment between the survey’s objective and questionnaire content, (ii) potential missing items, and (iii) the clarity of items and response options. Following this step, one item related to patient age groups was added, and minor comments provided on the clarity of some items were integrated.

The revised survey was subsequently pilot tested via* Sphinx Declic* (https://sphinxdeclic.com) amongst a convenience sample of five French chiropractors. They were contacted by email to complete the survey on *Sphinx Declic* and to provide feedback, using a table we had made available, on (i) the clarity of items and response options, (ii) any issues encountered while using *Sphinx Declic*, and (iii) the time taken to complete the survey. One item and five response options (related to four items) were clarified or modified in terms of presentation according to comments received; it took an average of 25 min to complete the survey.

### Data collection

Prior to distribution, the survey was promoted through an email sent to all licensed chiropractors by the AFC. The survey was distributed electronically using *Sphinx Declic* between February 20 and April 12, 2023. Chiropractors were invited to participate by email, highlighting the study’s purpose, content, and relevance for the profession. Potential participants were informed that answers were anonymous, and that participation was voluntary. Interested clinicians could access the survey using a link after giving their consent to participate. Enrolled clinicians could save their answers and complete the survey at a later date if they wished. Five reminders to complete the surveys were sent by the IFEC, AFC and AFCP, i.e., one to two reminders per association. Additionally, three reminders using *Sphinx Declic* were sent to non-responders and those who had partially completed the survey at ten days, four and five weeks.

### Data analysis

Data collected via* Sphinx Declic* were exported into Excel and de-identified. Descriptive statistics were performed in STATA 18. We computed means and standard deviations for continuous variables and percentages for categorical variables.

Representativeness of the results was estimated by comparing the demographic information (gender, age, and years since graduation) of survey participants with AFC members, using a two-sample Wilcoxon rank-sum (Mann Whitney) test for continuous data and Pearson chi-squared tests for categorical data. The level of significance was set to 0.05. Similarly, the demographics of participants who returned completed survey questionnaires were compared to those who provided incomplete questionnaires.

## Results

### Response rates and representativeness

The response rate was 46.4% (495 out of 1067 chiropractors invited). Of those, 386 (80%) completed the questionnaire, although some did not respond to all items. Three participants involved in at least one activity related to the chiropractic profession, but who were no longer in clinical practice, completed items 1–8. Both fully and partially completed questionnaires were included in the analysis. The number of respondents for each item is reported in the corresponding results tables (Tables 1, 2, 3, 4 and 5, Tables 1–13A in Additional file 2, and Table 1B in Additional file 3). The majority of respondents were members of the AFC (92.3%) and/or the AFCP (28.1%) (Table 1A, Additional file 2).

The study sample was comparable to AFC members for age (*p* = 0.86) and years since graduation (*p* = 0.32), but had a greater proportion of females (67.7% of respondents vs. 62.7% of AFC members; *p* = 0.045). Respondents who completed the survey were comparable to those provided incomplete survey data (n = 109) in terms of gender (*p* = 0.92), but were younger (*p* = 0.0007) and had graduated more recently (*p* = 0.008).

### Characteristics of participants

The mean age of participants was 34.9 years ± 9.7. The vast majority (94.5%) had graduated from IFEC and most others (4.8%) from a chiropractic program in the United States of America (USA) (Table 1A, Additional file 2); 81% of participants graduated in the past 15 years (Table 1). Just over one fifth (21.4%) reported having at least one additional university degree, including a Master’s degree (6.7%) or a PhD (1.2%) (Table 1). With the exception of three respondents, all were in private practice, with 22.7% of the participants reporting at least one other activity related to chiropractic in the previous 12 months. Among those, a majority (59.4%) taught in the IFEC undergraduate program and/or supervised chiropractic students in its outpatients’ clinics, and 8.3% indicated being involved in research in some capacity (Table 1). An equal proportion of respondents (49.8%) reported working full-time (≥ 35 h/week) or part-time (50.2%) (Table 2A, Additional file 2), with most of the working hours devoted to patient’s care (Table 3A, Additional file 2).

**Table 1:** Characteristics of participants

Years since graduation (n = 494)	% (n)
≤ 1–5 (2018–2023)	39.1% (n = 193)
6–10 (2013–2017)	26.5% (n = 131)
11–15 (2008–2012)	15.4% (n = 76)
16–20 (2003–2007)	6.3% (n = 31)
21–25 (1998–2002)	4% (n = 20)
26–30 (1993–1997)	2.8% (n = 14)
31–35 (1988–1992)	3.6% (n = 18)
> 35 (before 1988)	2.2% (n = 11)
University degrees (n = 495)	% (n)
Having one or several university degrees	21.4% (n = 106)
Bachelor	19.8% (n = 21)
Master’s degree	31.1% (n = 33)
PhD	5.7% (n = 6)
Medical degree	0.9% (n = 1)
Other university diploma*	59.4% (n = 63)
None or missing data**	78.6% (n = 389)
Activities related to the profession (n = 423)	% (n)
Yes	22.7% (n = 96)
Teaching and/or supervising clinicians	59.4% (n = 57)
Research	8.3% (n = 8)
Professional organization (e.g., administrative and/or managerial functions)	26% (n = 25)
Others (e.g., involved as association volunteer or in continuing education)	14.6% (n = 14)
None	77.3% (n = 327)
Private practice main activity in term of working hours/week? ^¥^ (n = 446)	% (n)
Yes	16.4% (n = 73)
No	5.2% (n = 23)
Not applicable (being exclusively in private practice)	78.5% (n = 350)
Engaged in at least one continuing education activity and types of continuing education activities in the past 12 months (n = 467)	% (n)
Yes	82.4% (n = 385)
University diploma course(s)	4.7% (n = 18)
Massive online open course(s)	18.7% (n = 72)
Scientific conference(s)	33.5% (n = 129)
Scientific literature (e.g., reading peer-reviewed articles, clinical guidelines)	34.3% (n = 132)
Seminar(s) on chiropractic practice	84.9% (n = 327)
Other(s) (e.g., taking part to a research project)	4.15% (n = 16)
No	13.3% (n = 62)
Not applicable^∫^	4.3% (n = 20)

*Diplôme Universitaire / Inter-Universitaire

**Participants were invited to skip this question when they did not have any university degree

^¥^Excluded participants not in clinical practice (n = 3)

^∫^Participants who graduated less than 12 months ago selected this answer

Over half of respondents (58.9%) reported seeing between 21 and 50 patients weekly, distributed as follows: 21–30 (19.7%), 31–40 (21.4%), and 41–50 (17.8%) (Table 4A, Additional file 2). The majority reported a mean number of new patients per week ranging between 4 and 9 (68.1%) (Table 4A, Additional file 2). Most participating chiropractors indicated seeing patients with acute or subacute symptoms within 0–2 days (61.3%) of the initial patient call, while patients with chronic symptoms were usually seen within 1–4 days (54.3%) (Table 5A, Additional file 2). Three quarters of respondents reported allocating over 40 min for an initial consultation, and 26–45 min for subsequent visits (Table 6A, Additional file 2).

About half (49.9%) participated in two or more continuing education activities in the past 12 months (Table 1). Continuing education activities included attending seminars focused on clinical practice (84.9%), reading scientific literature (e.g., peer-review articles, clinical guidelines) (34.3%), and attending scientific conferences (33.5%) (Table 1). Time spent by participants in continuing education activities is reported in Table 7A (Additional file 2).

### Practice characteristics

The majority of respondents (74.9%) worked in a single practice location (Table 8A, Additional file 2). Participants worked in either a multidisciplinary (27.4%) or a monodisciplinary setting (72.6%). Of those in monodisciplinary settings, 42.2% were solo practitioners and 54.1% worked with one or several other chiropractors (Table 8A, Additional file 2). Further, 17.5% reported doing out-of-office visits, such as patient houses and industries (Table 8A, Additional file 2).

Just over half of the respondents (53.5%) had their main practice location in one of four of the 18 French regions: *Ile-de-France* (20.1%), *Auvergne-Rhône-Alpes* (12%), *Occitanie* (12%), and *Nouvelle-Aquitaine* (9.5%). Participants with a second practice were most often located in these same regions or in *Bourgogne-Franche-Comté* and *Normandie* (Table 9A, Additional file 2). The size of the cities in which participants reported practicing is reported in Table 9A (Additional file 2).

Respondents offered services in French and in one or several other languages (63.1%), predominantly English (97.5%) (Table 8A, Additional file 2). Service charges for new patients usually ranged between 41 and 70 euros (90.75%), and for subsequent visits between 41 and 60 euros (83.2%) (Table 10A, Additional file 2).

About 95% of participating chiropractors reported feeling varying degrees of competition with other practitioners using manual therapy (e.g., osteopaths, physiotherapists), ranging from low (32.7%) to moderate (30.6%) and intense/very intense (31.65%) (Table 11A, Additional file 2).

### Characteristics of patients

#### Age groups

Consultations were more frequent among adults aged 25 to 64 years, followed by younger (15–24 years) and older (≥ 65 years) adults (Table 1B, Additional file 3). In contrast, pediatric age groups (≤ 14 years) predominantly fell within the 1–10% consultation range, with more than one fifth of respondents reporting not seeing patients under five years of age (Table 1B, Additional file 3).

#### Chief complaints

The two most common patient chief complaints were neck pain without arm pain and low back/pelvis pain without leg pain (mode = 11–20% range). Almost a quarter of respondents reported these two conditions as representing between 21 and 30% of their consultations. Mid-back pain with or without radiation and low back/pelvis with leg pain also represented a substantial proportion of chief complaints (Table 2).

**Table 2:** Percentages of patients seeking chiropractic care with various chief complaints

Chief complaint	None	1–10%	11–20%	21–30%	31–40%	41–50%	51–60%	61–100%
Headaches (tension-type and/or cervicogenic) (n = 376)	4.3% (n = 16)	57.45% (n = 216)	24.7% (n = 93)	8.5% (n = 32)	2.4% (n = 9)	0.8% (n = 3)	1.1% (n = 4)	0.8% (n = 3)
**Low back/pelvis pain without leg pain** (n = 385)	1% (n = 4)	12% (n = 46)	**30.9%** (n = 119)	**23.4%** (n = 90)	**15.6%** (n = 60)	7.8% (n = 30)	4.4% (n = 17)	4.9% (n = 19)
**Low back/pelvis pain with leg pain** (n = 380)	1.8% (n = 7)	**38.4%** (n = 146)	**32.6%** (n = 124)	**15%** (n = 57)	5% (n = 19)	3.4% (n = 13)	2.1% (n = 8)	1.5% (n = 6)
**Mid**-**back pain with or without irradiation** (n = 380)	1.8% (n = 7)	**45.3%** (n = 172)	**31.8%** (n = 121)	**11.3%** (n = 43)	3.7% (n = 14)	2.1% (n = 8)	2.4% (n = 9)	1.6% (n = 6)
**Neck pain without arm pain** (n = 379)	1.3% (n = 5)	**22.7%** (n = 86)	**37.5%** (n = 142)	**22.7%** (n = 86)	7.1% (n = 27)	3.2% (n = 12)	1.85% (n = 7)	3.7% (n = 14)
Neck pain with arm pain (n = 376)	4.3% (n = 16)	59.3% (n = 223)	25.8% (n = 97)	5.85% (n = 22)	2.7% (n = 10)	1.1% (n = 4)	0.8% (n = 3)	0.3% (n = 1)
Lower extremity disorder (n = 369)	4.9% (n = 18)	64.2% (n = 237)	17.9% (n = 66)	8.4% (n = 31)	1.1% (n = 4)	1.9% (n = 7)	1.1% (n = 4)	0.5% (n = 2)
Upper extremity disorder (n = 369)	2.4% (n = 9)	56.1% (n = 207)	25.75% (n = 95)	9.5% (n = 35)	3% (n = 11)	1.4% (n = 5)	0.5% (n = 2)	1.35% (n = 5)
Temporomandibular joint dysfunction syndrome (n = 360)	29.7% (n = 107)	60.3% (n = 217)	5.8% (n = 21)	2.5% (n = 9)	0.8% (n = 3)	0.3% (n = 1)	0.3% (n = 1)	0.3% (n = 1)
Prevention (secondary or tertiary of a musculoskeletal disorder) (n = 367)	11.2% (n = 41)	60.2% (n = 221)	17.4% (n = 64)	4.4% (n = 16)	2.7% (n = 10)	2.45% (n = 9)	0.3% (n = 1)	1.4% (n = 5)
Other musculoskeletal disorder (i.e., not listed above) (n = 349)	85.1% (n = 297)	12% (n = 42)	1.7% (n = 6)	0.3% (n = 1)	0	0.6% (n = 2)	0	0.3% (n = 1)
Non-musculoskeletal disorder (e.g., migraine) (n = 363)	19.8% (n = 72)	58.95% (n = 214)	12.7% (n = 46)	5.2% (n = 19)	1.4% (n = 5)	0.8% (n = 3)	0.55% (n = 2)	0.55% (n = 2)

In bold: the most frequent chief complaints for which patients seek care from French chiropractors, and the most frequent percentages of consultations represented by these complaints

### Patients’ management

#### Treatment modalities

Treatment modalities (e.g., high-velocity low-amplitude (HVLA) manipulations, cryotherapy), techniques/methods (e.g., *Mulligan* method, *McKenzie* method) and chiropractic systems (e.g., *Thompson* technique, *Activator Methods*) used are reported in Table 3. Most respondents reported delivering counseling on daily living habits (95.6%), using HVLA manipulations (93.5%), and patient education (86%). Passive myofascial techniques (86%) and exercises directed to the chief complaint (84.8%) were also commonly used. Few chiropractors (1.9%) reported having a room dedicated to exercise in their clinic(s), and about a quarter (27.2%) lend equipment such as elastic bands to their patients (Table 12A, Additional file 2).

**Table 3:** Percentage of chiropractors per treatment modality, technique, or chiropractic system used in their patients (n = 387)

Treatment modality / technique / chiropractic system	None	1–25%	26–50%	51–75%	76–100%
High-velocity and low-amplitude manipulation* (including Diversified and Gonstead techniques)	6.45% (n = 25)	10.1% (n = 39)	11.6% (n = 45)	**29.45%** (n = 114)	**42.4%** (n = 164)
Instrumentally assisted manipulation* (including Activator Methods)	13.2% (n = 51)	36.95% (n = 143)	19.9% (n = 77)	17.3% (n = 67)	12.7% (n = 49)
Mechanically assisted manipulation* (including Thompson and Hole In One techniques)	22.2% (n = 86)	33.3% (n = 129)	18.6% (n = 72)	16.3% (n = 63)	9.6% (n = 37)
Spinal traction (including Cox technique)	38% (n = 147)	39% (n = 151)	13.2% (n = 51)	7% (n = 27)	2.8% (n = 11)
Vertebral mobilization (e.g., Mc Kenzie, Mulligan and Maitland techniques)	36.95% (n = 143)	36.4% (n = 141)	14.2% (n = 55)	8% (n = 31)	4.4% (n = 17)
Orthopedic blocking	27.4% (n = 106)	23.5% (n = 91)	14.5% (n = 56)	15.5% (n = 60)	19.1% (n = 74)
**Passive myofascial techniques** (e.g., stretching, massage, Trigger Point, Graston and Dry Needling techniques)	13.95% (n = 54)	**24%** (n = 93)	17.8% (n = 69)	18.6% (n = 72)	**25.6**% (n = 99)
Physical therapy modalities (e.g., transcutaneous electric nerve stimulation, cryotherapy)	75.45% (n = 292)	19.6% (n = 76)	2.8% (n = 11)	1.3% (n = 5)	0.8% (n = 3)
Taping (e.g., Kinesio taping technique)	31.3% (n = 121)	52.45% (n = 203)	12.4% (n = 48)	2.6% (n = 10)	1.3% (n = 5)
Strapping and/or advices related to orthotic devices (e.g., bracing)	57.1% (n = 221)	37.5% (n = 145)	3.6% (n = 14)	1% (n = 4)	0.8% (n = 3)
**Exercises directed to chief complaint** (supervised or not, e.g., stretching, strengthening)	15.2% (n = 59)	**23.5%** (n = 91)	**22.7%** (n = 88)	18.9% (n = 73)	19.6% (n = 76)
Applied kinesiology	49.6% (n = 192)	22% (n = 85)	7.2% (n = 28)	5.4% (n = 21)	15.8% (n = 61)
Bio energetic synchronization technique	74.4% (n = 288)	19.4% (n = 75)	3.1% (n = 12)	1.55% (n = 6)	1.55% (n = 6)
Network technique	81.1% (n = 314)	17.3% (n = 67)	1% (n = 4)	0.5% (n = 2)	0
Sacrooccipital technique	35.65% (n = 138)	18.9% (n = 73)	13.2% (n = 51)	10.3% (n = 40)	22% (n = 85)
**Information specific to the chief complaint** (e.g., its natural course, reinsurance)	13.95% (n = 54)	**16%** (n = 62)	11.6% (n = 45)	15.2% (n = 59)	**43.15%** (n = 167)
**Counseling on daily living habits** (e.g., physical activity, nutrition, sleep)	4.4% (n = 17)	12.7% (n = 49)	16.3% (n = 63)	**20.4%** (n = 79)	**46.25%** (n = 179)
Other(s)	87.85% (n = 340)	6.45% (n = 25)	1.55% (n = 6)	1.3% (n = 5)	2.8% (n = 11)

^*^Modality that can be delivered at the spine and/or extremities

In bold: the most frequently reported therapeutic modalities used by French chiropractors, and the main patient percentage ranges for which these modalities are applied

#### Referrals

Participating chiropractors reported referring patients “sometimes” (1–3 times/month) or “often-routinely” (at least 1/week) to general practitioners (50.4% and 21% respectively), physiotherapists (“sometimes” 41.5%; “often-routinely” 23.7%), and podiatrists (“sometimes” 36.8%; “often-routinely” 12.7%) (Table 4).

**Table 4:** Frequency of chiropractors referring to various healthcare professionals during de past 12 months

Health care professional	Never	Rarely (< 1/month)	Sometimes (1–3/month)	Often-Routinely (> 1/week)
**General practitioner** (n = 367)	1.9%(n = 7)	26.7% (n = 98)	50.4% (n = 185)	21% (n = 77)
Pediatrician (n = 349)	50.1% (n = 175)	34.1% (n = 119)	12.9% (n = 45)	2.9% (n = 10)
Dentist (n = 359)	26.7% (n = 96)	43.2% (n = 155)	24% (n = 86)	6.1% (n = 22)
Doctor specialized in sport medicine (n = 346)	67.05% (n = 232)	23.4% (n = 81)	7.5% (n = 26)	2% (n = 7)
Doctor specialized in physical and rehabilitation medicine (n = 341)	73.6% (n = 251)	20.2% (n = 69)	5% (n = 17)	1.2% (n = 4)
Neurologist (n = 357)	30.5% (n = 109)	49% (n = 175)	19.6% (n = 70)	0.8% (n = 3)
Rheumatologist (n = 355)	28.45% (n = 101)	49.3% (n = 175)	20.85% (n = 74)	1.4% (n = 5)
Gynecologist ± obstetrician (n = 349)	53.6% (n = 187)	32.95% (n = 115)	12.3% (n = 43)	1.1% (n = 4)
Occupational physician (n = 349)	83.7% (n = 292)	14% (n = 49)	2% (n = 7)	0.3% (n = 1)
Orthopedic surgeon (n = 354)	40.1% (n = 142)	43.2% (n = 153)	15% (n = 53)	1.7% (n = 6)
Neurosurgeon (n = 356)	44.9% (n = 160)	41% (n = 146)	11.8% (n = 42)	2.2% (n = 8)
**Physiotherapist** (n = 359)	11.7% (n = 42)	23.1% (n = 83)	41.5% (n = 149)	23.7% (n = 85)
Midwife/maieutician (n = 347)	53.3% (n = 185)	27.7% (n = 96)	13.5% (n = 47)	5.5% (n = 19)
**Podiatrist** (n = 361)	15.2% (n = 55)	35.2% (n = 127)	36.8% (n = 133)	12.7% (n = 46)
Chiropractor (n = 346)	27.5% (n = 95)	50% (n = 173)	18.2% (n = 63)	4.3% (n = 15)
Osteopath (n = 349)	76.2% (n = 266)	22.6% (n = 79)	0.9% (n = 3)	0.3% (n = 1)
Other(s) (whether regulated or not) (n = 321)	43.3% (n = 139)	28.35% (n = 91)	18.7% (n = 60)	9.7% (n = 31)

In bold: the healthcare professionals to whom French chiropractors most frequently report referring patients

The most common sources of patient referrals (“sometimes” to “often-routinely”) were from general practitioners (41.3% and 10.4% respectively), physiotherapists (27.8% and 8.1% respectively), midwifes (15.7% and 9.2% respectively), and other chiropractors (19.5% and 4.2% respectively) (Table 5). With the exception of dentists, over 70% of respondents reported never receiving referrals from the medical specialists (Table 5).

**Table 5:** Frequency of various healthcare professionals referring to chiropractors during the past 12 months

Health care professional	Never	Rarely (< 1/month)	Sometimes (1–3/month)	Often-Routinely (> 1/week)
**General practitioner** (n = 366)	13.7% (n = 50)	34.7% (n = 127)	41.3% (n = 151)	10.4% (n = 38)
Pediatrician (n = 355)	73% (n = 259)	17.75% (n = 63)	7.9% (n = 28)	1.4% (n = 5)
Dentist (n = 363)	54.3% (n = 197)	29.75% (n = 108)	13.2% (n = 48)	2.75% (n = 10)
Sports doctor (n = 356)	79.8% (n = 284)	17.1% (n = 61)	2.5% (n = 9)	0.6% (n = 2)
Doctor specialized in physical and rehabilitation medicine (n = 356)	87.1% (n = 310)	9.8% (n = 35)	2.5% (n = 9)	0.6% (n = 2)
Neurologist (n = 359)	78.6% (n = 282)	16.2% (n = 58)	5% (n = 18)	0.3% (n = 1)
Rheumatologist (n = 359)	70.2% (n = 252)	22.8% (n = 82)	5.85% (n = 21)	1.1% (n = 4)
Gynecologist ± obstetrician (n = 355)	87.9% (n = 312)	7.3% (n = 26)	4.5% (n = 16)	0.3% (n = 1)
Occupational physician (n = 355)	88.7% (n = 315)	9.9% (n = 35)	1.4% (n = 5)	0
Orthopedic surgeon (n = 359)	77.2% (n = 277)	17.55% (n = 63)	4.5% (n = 16)	0.8% (n = 3)
Neurosurgeon (n = 358)	80.7% (n = 289)	14.8% (n = 53)	3.35% (n = 12)	1.1% (n = 4)
**Physiotherapist** (n = 360)	26.9% (n = 97)	37.2% (n = 134)	27.8% (n = 100)	8.05% (n = 29)
**Midwife/maieutician** (n = 357)	49.6% (n = 177)	25.5% (n = 91)	15.7% (n = 56)	9.2% (n = 33)
Chiropractor (n = 359)	27.9% (n = 100)	48.5% (n = 174)	19.5% (n = 70)	4.2% (n = 15)
Osteopath (n = 353)	54.7% (n = 193)	36% (n = 127)	8.2% (n = 29)	1.1% (n = 4)
Other(s) (whether regulated or not) (n = 333)	51.65% (n = 172)	20.1% (n = 67)	18% (n = 60)	10.2% (n = 34)

In bold: the healthcare professionals most frequently reported as referring patients to French chiropractors

When diagnostic imaging (ultrasound, X-rays, CT scan or MRI) was deemed necessary, the majority of the respondents (between 88.6.1% and 96.5%) reported referring patients to their general practitioner, most often without a referral letter (between 58.1% and 71.8%) (Table 13A, Additional file 2). Few participating chiropractors reported referring patients directly to radiologists (3.5%-11.4%) or performing musculoskeletal ultrasound themselves (1.6%) (Table 13A, Additional file 2).

## Discussion

This study provides the first comprehensive description of French chiropractors’ profiles and activities, patients seeking chiropractic care, and patterns of interprofessional referrals. Just over a quarter of participating chiropractors worked in a multidisciplinary setting. They reported referring patients to other healthcare professionals, particularly general practitioners, more frequently than they received referrals from them. Middle-aged adults were the most common patients seeking care, and spinal pain was the main chief complaint. Treatment modalities generally consisted of manual therapy not restricted to HVLA manipulations, advice, patient education, and therapeutic exercises.

### Characteristics of chiropractors

The characteristics of our sample were broadly comparable to those of AFC members in terms of age and years since graduation, although the proportion of females was significantly higher (67.7% vs. 62.7%). This possible overrepresentation may partly explain the lower mean number of working hours per week and the number of patients seen weekly in our sample. Previous studies have reported gender-based differences in these two parameters, whereas no such differences have been observed for other aspects of practice, such as therapeutic modalities used or patient characteristics [22, 23]. The mean age of participants and their years in practice distribution were generally consistent with the demographic profile of the profession in France, where chiropractic remains relatively young. Since the establishment of the IFEC program in 1984, the number of chiropractic graduates per year has steadily increased, with an average growth of 83.2% between 2007 and 2012, and 67.8% between 2012 and 2024.

Consistent with trends observed in other European countries [24], approximately one in five respondents reported holding at least one university degree in addition to their chiropractic diplomas. Most of the Master’s (6.7%) and PhD (1.2%) degrees were obtained from the University of Paris-Saclay in France, with which the IFEC has established partnerships to facilitate access to academic courses. The vast majority of respondents had engaged in at least one continuing education activity in the 12 months preceding the survey. While continuing education requirements for chiropractors are still awaiting clarification by the legislator, it appears that part of the profession is already engaged in a continuing education dynamic, with over 70% of activities attended likely involving evidence-based education (e.g., scientific conferences, reading of scientific literature, or university diplomas or courses). Although research capacities within the French chiropractic profession remain limited [18], the use of evidence-based information appears to inform clinical practice among participating clinicians.

Approximately half of chiropractors reported working full-time, a trend similar to other countries [14, 20, 25–27]. The proportion working less than 21 h per week was comparable to the USA and South Africa [26, 27], but more than twice as high as in Switzerland and Denmark [14, 20]. Factors contributing to part-time practice may include the relatively high proportion of younger female practitioners [22, 23, 27] and involvement in related activities such as teaching [14, 27]. Adams et al. [21] proposed to investigate alternative explanations, including intra- and interprofessional competition, which could limit the number of patients seen. In the present survey, 62.3% of respondents reported experiencing medium to very intense competition with other practitioners using manual therapy supporting the possibility that competition influences practice patterns. The number of patients seen weekly varied considerably among respondents, showing both similarities (e.g., in the mean number of new patients per week) [14, 26], and differences [14, 21, 28, 29] compared with other countries. In comparison with recent studies conducted among chiropractors in other countries, i.e., Switzerland [14], Australia [21], Canada [28], and USA [29], participating chiropractors appeared to see fewer patients per week. This may partly be explained by longer visit durations, particularly for follow-up appointments [14], but it could also reflect differences in the organization of healthcare systems.

### Characteristics of patients and treatment modalities used

Consistent with previous research [10, 14, 26, 28, 29], most patients seeking chiropractic care were adults between the ages of 25–64 years, followed by older adults (≥ 65 years). Pediatric patients, particularly children under the age of five years, accounted for only a small proportion of chiropractic visits. As reported elsewhere, the majority of consultations were for musculoskeletal conditions, primarily back and neck pain, suggesting that both patients and healthcare professionals referring patients generally recognize chiropractors’ scope of practice [10, 14, 21, 26, 28, 29]. The reported use of therapeutic modalities suggests that respondents often combined modalities as recommended by clinical practice guidelines [5–8].

### Interprofessional referrals

Most participating chiropractors reported regularly referring patients to various healthcare providers, particularly general practitioners and physiotherapists, yet they reported receiving fewer referrals. In the French healthcare system, access to most conventional healthcare providers (e.g., physiotherapists and medical specialists) and the level of reimbursement for their services depend, at least in part, on a referral from a general practitioner. This likely explains why respondents most frequently reported referring patients to general practitioners. Although French chiropractors are first-contact healthcare providers, they are not yet fully integrated within the conventional healthcare system. As a result, chiropractic care remains outside the national health insurance coverage and is reimbursed only by certain private health insurers, a situation that likely hindering its integration into established care pathways. In this context, general practitioners are more likely to refer patients, for example, to physiotherapists, whose services are almost fully covered by the national health insurance system.

Several barriers may further limit interprofessional collaboration with chiropractors, including limited awareness of the chiropractic profession and insufficient knowledge of its scope of practice, educational standards, and training requirements [15, 30, 31]. Embedding chiropractic education within public health faculties, as seen in Switzerland and Denmark, appears to facilitate the integration of chiropractors into the healthcare system [14, 15]. Such integration may strengthen interprofessional collaboration by fostering early professional relationships and enhancing the perceived credibility of chiropractic training among other healthcare professionals [15, 32, 33]. In a recent survey conducted among Swiss chiropractors, 71% of respondents reported receiving at least one patient referral per week from a general practitioner, compared to 10.4% in the present survey [14]. The results of a 2020 survey among Danish chiropractors also suggest a higher rate of patient referrals from general practitioners [15].

Working in multidisciplinary settings can also facilitate interprofessional collaboration [32, 33]. In our survey, 27% of respondents reported working in such settings. However, we did not explore whether their colleagues belonged to the mainstream French healthcare system. Further research exploring the barriers and facilitators influencing both patients’ access to chiropractic care and chiropractors’ referrals to and from other healthcare professionals could provide valuable insights to support more effective interprofessional collaboration.

### Strengths and limitations

Although no exhaustive list of licensed chiropractors exists in France, and only two of the six French chiropractic associations along with the IFEC contributed to the recruitment strategy, approximately 77.2% of the profession was invited to participate. The response rate was reasonably high (46.4%), with 80% of those returning completed surveys. With few exceptions [14, 20], similar surveys have reported comparable or lower response rates [21, 26, 28]. Nonetheless, this study has several limitations. The survey was long, which likely contributed to participant dropouts [34]. In fact, 75% of the dropouts occurred when moving from one section to the next, suggesting participant fatigue. Together, the recruitment strategy and the response rate may have introduced selection bias. Additionally, respondents who completed the surveys were younger and had graduated more recently than those who provided incomplete surveys. Still, the consistency between several of our findings and those of similar job analyses conducted in other countries suggests that the present survey provides a realistic portrait of the French chiropractic profession, at least for patient age groups, reasons for seeking chiropractic care, and commonly used treatment modalities.

Self-reported cross-sectional surveys may be prone to recall and social desirability biases. For example, some items may be perceived as sensitive (e.g., the intensity of the perceived competition with other healthcare providers using manual therapy). However, the results obtained for such items suggest that participants provided honest responses. Finally, some answers were collected in the form of ranges rather than precise values, allowing only for rough estimates. Future prospective studies or studies based on patient records would provide more accurate data on some aspects of the chiropractic profession in the French territories.

## Conclusion

The chiropractic workforce appears predominantly composed of practitioners who have graduated in the past 15 years. With few exceptions, practice and patient profiles were similar to those described in other countries and aligned with the legal scope of practice of French chiropractors. In the context of increasing unmet healthcare needs of people with MSDs, further research to help understand how French chiropractors could be better integrated into the healthcare system in France would be of interest.

## Supplementary Information

Below is the link to the electronic supplementary material.

Supplementary Material 1

Supplementary Material 2

Supplementary Material 3

## Data Availability

The datasets analyzed during the current study are available from the corresponding author on reasonable request.

## Abbreviations

AFC: Association Française de Chiropraxie
AFCP: Association Française de Chiropraxie Pédiatrique
HVLA: High-velocity and low-amplitude
IFEC: Institut Franco-Européen de Chiropraxie
MSDs: Musculoskeletal disorders
USA: United States of America
