# Supplementary material for: Contemporary profiles and professional activities of French chiropractors: a national survey
Source: Chiropr Man Therap. 2025 Oct 13;33:43. doi: 10.1186/s12998-025-00602-2 (PMC12516846; doi:10.1186/s12998-025-00602-2)
Supplement: Supplementary file 1 — Supplementary Material 1 [file 12998_2025_602_MOESM1_ESM.docx]

**Additional file 1**: Survey instrument

Original version (French)

NB : A non-cross cultural adapted translated version to English (using artificial intelligence) is available on pages 17 to 29.

**Profils et activités des chiropracteurs en France**.

Merci d’avoir accepté de compléter l’enquête « Profils et activités des chiropracteurs en France » ; votre contribution est essentielle !

En fonction de votre statut (ex. de votre année de diplôme, que vous ayez ou non une ou des activités en lien avec la profession autres que celle de cabinet), il est possible que vous soyez ponctuellement redirigé(e) vers une question additionnelle ou, au contraire, vers une question dont la numérotation ne suit pas immédiatement celle de la dernière question ou du dernier item complété(e). Ainsi, ne prêtez pas attention à la numérotation des items et questions, celle-ci servira de repère lors de l’analyse des données.

Si besoin, l’enquête peut être complétée en plusieurs fois. Vos réponses sont sauvegardées dès que vous passez à la page suivante du questionnaire. Ainsi, pensez à passer à la page suivante même si vous n’avez pas terminé de répondre à l’ensemble des questions d’une page, ce afin de ne pas perdre vos réponses. De la même façon, si vous pensez être interrompu(e) 20 minutes ou plus et donc être inactif / inactive sur une page de l’enquête pendant ce temps, sauvegardez vos réponses en passant à la page suivante afin de pouvoir y revenir sans perdre les réponses déjà apportées.

Enfin, dans le cas où vous auriez été contacté(e) via différentes adresses mail, complétez une seule fois l’enquête et, si vous la complétez en plusieurs fois, faites-le toujours à partir de la même adresse mail.

**I Caractéristiques individuelles**

**1 - Indiquez votre âge**: … … … … …

**2 - Indiquez votre genre** :

Féminin

Masculin

Autre

**3 - Indiquez l’année d’obtention de votre diplôme de chiropracteur**: … … … … …

**4 - Indiquez l’établissement dans lequel vous avez obtenu votre diplôme de chiropracteur** :

Institut Franco-Européen de Chiropraxie

Anglo-European College of Chiropractic

Cleveland Chiropractic College

Life Chiropractic College West

Life University

Logan Chiropractic College

Los Angeles College of Chiropractic

National University of Health Sciences

New York Chiropractic College

Palmer College of Chiropractic

Palmer College of Chiropractic West

Sherman Chiropractic College

Texas Chiropractic College

Western States Chiropractic College

Autre (préciser) : … … … … …

**5** - **Dans le cas où vous êtes titulaire d’un ou plusieurs autres diplômes que celui de chiropracteur, indiquez le- ou lesquels** **en précisant la ou les spécialisations** :

- Ce(s) niveau(x) d’éducation peut/peuvent avoir été atteint(s) avant ou après l’obtention du titre de chiropracteur.

Licence

**Spécialisation(s)** : … … … … …

Diplôme(s) universitaire(s) (DU) / Diplôme(s) interuniversitaire(s) (DIU) :

**Spécialisation(s)** : … … … … …

Master

**Spécialisation(s)** : … … … … …

Doctorat

**Spécialisation(s)** : … … … … …

Autre(s) (préciser) : … … … … …

**6 - Indiquez la ou les associations professionnelles françaises en relation avec la chiropraxie dont vous êtes membre**:

Aucune

Association Française de Chiropraxie.

Syndicat National des Chiropracteurs.

Conseil Français de Chiropraxie du Sport.

Association Française de Chiropraxie Pédiatrique.

Association Française de Chiropraxie Animale.

Association Française pour l’Histoire de la Chiropratique en France.

Autre(s) (préciser) : …………………

**7 - En tant que chiropracteur(e), que vous soyez ou non en exercice** (c’est à dire que vous ayez ou non une activité clinique)**, indiquez la ou les activités** (rémunérées ou non) **relatives à la chiropraxie dans la- ou lesquelles vous avez été impliqué(e) durant les 12 derniers mois**:

Aucune

Enseignement (dans un cadre de formation initiale) et/ou encadrement pédagogique

Recherche (en tant qu’investigateur et/ou co-auteur d’article(s) scientifique(s))

Administratives/de gestion et/ou de management (ex. au sein de l’Institut Franco-Européen de Chiropraxie / de l’Association Française de Chiropraxie / du Fond de Dotation pour la Recherche en Chiropratique)

Autre(s) (préciser) : … … … … …

**8 - Indiquez la situation qui est la vôtre au moment où vous complétez l’enquête** :

Je n’exerce plus en tant que chiropracteur(e) mais ai conservé une ou des activités en lien avec la chiropraxie en France.

J’exerce en tant que chiropracteur(e) à temps plein ou à temps partiel sur le territoire français.

**II Caractéristiques professionnelles : temps de travail hebdomadaire et activités en lien avec la chiropraxie**

**Concernant les questions et items ci-dessous, si vous avez plus d’un lieu d’exercice, l’ensemble de votre activité est à prendre en compte pour y répondre**.

**9** - **Indiquez le temps de travail hebdomadaire que vous consacrez en moyenne à l’exercice de la chiropraxie** (comprenant la prise en charge des patients ainsi que la gestion de votre activité) : … … … … …

**10** - **Dans le cadre de votre exercice de la chiropraxie, comment se répartissent, en pourcentage de temps de travail hebdomadaire, les activités listées ci-dessous**?

- Le total des proportions indiquées doit être égal à 100%.

|  | **1-25%** | **26-50%** | **51-75%** | **76-100%** |
| --- | --- | --- | --- | --- |
| **Prise en charge des patients** (comprenant anamnèse, examen, traitement, information/conseils et tenue du dossier patient, communication interprofessionnelle) |  |  |  |  |
| **Gestion de l’activité** (ex. administratif - y compris la comptabilité et la gestion du personnel si vous en avez -, communication) |  |  |  |  |

**11 - En moyenne, combien de temps consacrez-vous à un nouveau patient** (première consultation) ?

≤ 5min

6-10 min

11-15 min

16-20 min

21-30 min

31-45 min

46-60 min

> 60min

**12 - En moyenne, combien de temps consacrez-vous à un patient lors d’une consultation de suivi**?

≤ 5min

6-10 min

11-15 min

16-20 min

21-30 min

31-45 min

46-60 min

> 60min

**13 - En moyenne, combien de consultations effectuez-vous personnellement par semaine** ?

≤ 10

11-20

21-30

31-40

41-50

51-60

61-70

71-80

81-90

91-100

101-110

≥ 111

**14 - Indiquez le nombre moyen de nouveaux patients que vous recevez personnellement par semaine**:

0

1-3

4-6

7-9

10-12

13-15

> 15

**15** - **En moyenne, dans quel délai recevez-vous un patient présentant une symptomatologie aiguë ou subaiguë** (d’une durée inférieure à 3 mois) ?

Le jour même

1-2 jours

3-4 jours

5-7 jours

1-2 semaines

> 2 semaines

**16** - **En moyenne, dans quel délai recevez-vous un patient présentant une symptomatologie chronique** (d’une durée supérieure à 3 mois) ?

Le jour même

1-2 jours

3-4 jours

5-7 jours

1-2 semaines

> 2 semaines

**17 - Dans le cas où vous auriez différentes activités rémunérées en lien avec la chiropraxie, votre activité en clinique (c’est-à-dire l’exercice de la chiropraxie) représente-t-elle votre activité principale** (en termes de temps de travail hebdomadaire, non en termes de rémunération) ?

- Sélectionnez « Non applicable » dans le cas où votre activité clinique occupe 100% de votre temps de travail hebdomadaire.

Non-applicable

Oui

Non

**18 - Indiquez le ou les types d’actions de formation continue auxquelles vous avez participées durant les 12 derniers mois** :

- Sélectionnez « Non applicable » dans le cas où vous êtes diplômé(e) chiropracteur(e) depuis moins de 12 mois.

Non-applicable

Je n’ai participé à aucune action de formation continue au cours des 12 derniers mois.

Formation universitaire diplômante (Licence, DU/DIU, Master, Doctorat)

Séminaire(s) portant sur des sujets chiropratiques ou se rapportant à l’exercice de la chiropraxie (en présentiel et/ou en ligne)

Conférence(s) scientifique(s) et/ou de vulgarisation scientifique (en présentiel et/ou en ligne)

Cours en ligne de type *massive online open course (MOOC)* / formation en ligne ouverte à tous (FLOT)

Lecture d’articles scientifiques et/ou de travaux scientifiques (ex. recommandations de bonne pratique, thèse de doctorat)

Autre(s) (ex. participation à un/des projets de recherche) (préciser) : … … … … …

**19 - Indiquez le nombre d’heures que vous avez consacré en moyenne à des actions de formation continue durant les 12 derniers mois** :

1-10 heures

11-20 heures

21-30 heures

31-40 heures

41-50 heures

51-60 heures

61-70 heures

71-80 heures

81-90 heures

91-100 heures

> 100 heures

**III Pratique clinique : patients, examens complémentaires, modalités thérapeutiques/techniques et pluridisciplinarité**

**20 – En moyenne chaque semaine et sur la base de votre activité en général** (c’est à dire incluant l’ensemble de vos lieux d’exercice)**, quelle proportion de patients vous consultent pour les motifs proposés ci-dessous (en tant que motif principal de consultation) ?**

- Si vous ne prenez en charge aucun patient pour un ou plusieurs motifs proposés ci-dessous, cochez "Aucun patient".
- Le total des proportions indiquées doit être égal à 100%

|  | **Aucun patient** | **1-10%** | **11-20%** | **21-30%** | **31-40%** | **41-50%** | **51-60%** | **61-70**  **%** | **71-80**  **%** | **81-90%** | **91-100%** |
| --- | --- | --- | --- | --- | --- | --- | --- | --- | --- | --- | --- |
| **Lombalgie mécanique / douleur de la région lombo-pelvienne, sans irradiation** au(x) membre(s) inférieur(s) |  |  |  |  |  |  |  |  |  |  |  |
| **Lombalgie mécanique / douleur de la région lombo-pelvienne, avec irradiation** au(x) membre(s) inférieur(s) |  |  |  |  |  |  |  |  |  |  |  |
| **Dorsalgie mécanique avec ou sans irradiation** |  |  |  |  |  |  |  |  |  |  |  |
| **Cervicalgie mécanique sans irradiation** au(x) membre(s) supérieur(s) |  |  |  |  |  |  |  |  |  |  |  |
| **Cervicalgie mécanique avec irradiation** au(x) membre(s) supérieur(s) |  |  |  |  |  |  |  |  |  |  |  |
| **Céphalées de tension** et/ou **cervicogéniques** |  |  |  |  |  |  |  |  |  |  |  |
| **Trouble musculosquelettique du membre supérieur** |  |  |  |  |  |  |  |  |  |  |  |
| **Trouble musculosquelettique du membre inférieur** |  |  |  |  |  |  |  |  |  |  |  |
| **Syndrome algo-dysfonctionnel de l’appareil manducateur** |  |  |  |  |  |  |  |  |  |  |  |
| **Prévention secondaire et/ou tertiaire* de trouble(s) musculosquelettique(s)** |  |  |  |  |  |  |  |  |  |  |  |
| **Autre(s) trouble(s) musculosquelettique(s)** (non mentionné(s) ci-dessus) |  |  |  |  |  |  |  |  |  |  |  |
| **Trouble(s) non-musculosquelettique(s)** (ex. migraine) |  |  |  |  |  |  |  |  |  |  |  |

* La Haute Autorité de Santé (HAS) définit la prévention comme suit : « La prévention consiste à éviter l'apparition, le développement ou l'aggravation de maladies ou d'incapacités.
Sont classiquement distinguées la prévention primaire qui agit en amont de la maladie (ex. : vaccination, action sur les facteurs de risque), la prévention secondaire qui agit à un stade précoce de son évolution (dépistages*, ex. : dépistage de scoliose*), et la prévention tertiaire qui agit sur les complications et les risques de récidive *(ex. : suivi de patients lombalgiques chroniques ou récurrentes)*. »

**21 - Sur la base de votre activité en général** (c’est à dire incluant l’ensemble de vos lieux d’exercice)**, indiquez dans le tableau ci-dessous la proportion de patients pris en charge en fonction de leur catégorie d’âges**:

- Si vous ne rencontrez pas dans votre pratique une ou plusieurs des catégories d’âges proposées ci-dessous, sélectionnez « Aucun patient ».
- Le total des proportions indiquées doit être égal à 100%.

|  | **Aucun patient** | **1-10%** | **11-20%** | **21-30%** | **31-40%** | **41-50%** | **51-60%** | **61-70%** | **71-80%** | **81-90%** | **91-100%** |
| --- | --- | --- | --- | --- | --- | --- | --- | --- | --- | --- | --- |
| **< 6 mois** |  |  |  |  |  |  |  |  |  |  |  |
| **6 mois - 5 ans** |  |  |  |  |  |  |  |  |  |  |  |
| **6 - 14 ans** |  |  |  |  |  |  |  |  |  |  |  |
| **15 - 24 ans** |  |  |  |  |  |  |  |  |  |  |  |
| **25 - 39 ans** |  |  |  |  |  |  |  |  |  |  |  |
| **40 - 64 ans** |  |  |  |  |  |  |  |  |  |  |  |
| **≥ 65 ans** |  |  |  |  |  |  |  |  |  |  |  |

**22 - Indiquez la situation correspondant le mieux à vos habitudes lorsque des clichés radiographiques sont indiqués** :

Lorsqu’un besoin de clichés radiographiques se présente, le plus souvent, j’en fais la demande directement à un radiologue.

Lorsqu’un besoin de clichés radiographiques se présente, le plus souvent, j’invite le patient à en faire la demande auprès de son médecin généraliste (sans courrier à destination de ce dernier).

Lorsqu’un besoin de clichés radiographiques se présente, le plus souvent, j’invite le patient à en faire la demande auprès de son médecin généraliste (avec courrier à destination de ce dernier).

**23 - Indiquez la situation correspondant le mieux à vos habitudes lorsqu’une échographie est indiquée** :

|  | **Le plus souvent, je réalise moi-même cet examen** | **Le plus souvent, je réfère le patient directement à un radiologue** | **Le plus souvent, je réfère le patient vers son médecin généraliste (sans courrier)** | **Le plus souvent, je réfère le patient vers son médecin généraliste (avec courrier)** |
| --- | --- | --- | --- | --- |
| **Echographie musculosquelettique** |  |  |  |  |
| **Echographie non-musculosquelettique** | Non applicable |  |  |  |

**24 - Indiquez la situation correspondant le mieux à vos habitudes lorsqu’un scanner ou une IRM est indiqué(e)**:

Lorsqu’un besoin de scanner ou d’IRM se présente, le plus souvent, j’en fais la demande directement à un radiologue.

Lorsqu’un besoin de scanner ou d’IRM se présente, le plus souvent, j’invite le patient à en faire la demande auprès de son médecin généraliste (sans courrier à destination de ce dernier).

Lorsqu’un besoin de scanner ou d’IRM se présente, le plus souvent, j’invite le patient à en faire la demande auprès de son médecin généraliste (avec courrier à destination de ce dernier).

**25 - Pour chaque modalité thérapeutique et technique ou approche, indiquez l’intervalle correspondant le mieux au pourcentage de patients pour lesquels vous y avez recours :**

- Si vous n’utilisez pas une ou plusieurs modalités thérapeutiques et/ou techniques/approches listées ci-dessous, ne cochez rien pour la ou les lignes concernées.

|  | **1-25%** | **26-50%** | **51-75%** | **76-100%** |
| --- | --- | --- | --- | --- |
| **Manipulations vertébrales/articulaires à haute vélocité et faible amplitude** (*high velocity and low amplitude*, incluant les techniques *Diversified* et *Gonstead*) |  |  |  |  |
| **Manipulations instrumentales**, incluant la technique *Activator* |  |  |  |  |
| **Manipulations mécaniquement assistées**, incluant la technique *Thompson* |  |  |  |  |
| **Traction vertébrale**, incluant la technique de flexion-distraction *Cox* et la traction vertébrale manuelle |  |  |  |  |
| **Mobilisations vertébrales/articulaires**, incluant les mobilisations de types *McKenzie*, *Mulligan, Maitland* |  |  |  |  |
| **Blocs orthopédiques** (utilisation de blocs hors du cadre de techniques ou approches spécifiques de type *Technique Sacrooccipitale*) |  |  |  |  |
| **Technique Sacrooccipitale** (*Sacrooccipital technique*) |  |  |  |  |
| **Kinésiologie Appliquée** (*Applied kinesiology*) |  |  |  |  |
| **Technique *Network*** (*Network technique*) |  |  |  |  |
| **Technique de *Synchronisation Bio Energétique*** (*Bio Energetic Synchronization Technique*) |  |  |  |  |
| **Techniques myofasciales passives**, c’est à dire appliquées par le chiropracteur, visant les tissus mous (ex. *trigger point/pressions ischémiques*, *Dry Needling*, *Graston*, étirements passifs, massage) |  |  |  |  |
| **Modalités de thérapie physique** (e*x*. électrostimulation de type TENS, laser, application de chaud et/ou utilisation de la cryothérapie) |  |  |  |  |
| **Taping** (*ex. Kinésio Taping*) |  |  |  |  |
| **Strapping et/ou conseils relatifs à des moyens de contention** |  |  |  |  |
| **Exercices à visée thérapeutique, supervisés ou non** (ex. renforcement musculaire, proprioception, étirement) |  |  |  |  |
| **Délivrance d’informations relatives au motif de consultation du patient lui permettant d’être acteur de sa prise en charge** (ex. caractère bénin et habituellement récurrent de la lombalgie, alternatives thérapeutiques disponibles et bénéfices/risques attendus de ces dernières) |  |  |  |  |
| **Conseils d’ordre général, relatifs au mode de vie** (ex. sommeil, nutrition, activité physique) |  |  |  |  |
| **Autre(s)** |  |  |  |  |

**26 - Vous arrive-t-il de mettre à disposition de vos patients du matériel de réhabilitation**/**rééducation fonctionnelle** (ex. élastiques, balle/rouleau de massage, coussin de proprioception) ?

Oui

Non

**27 - Disposez-vous d’une pièce dédiée à la réhabilitation**/**rééducation fonctionnelle au sein de l’un ou de plusieurs de vos lieux d’exercice**?

- Dans le cas où vous êtes chiropracteur(e) exerçant uniquement au domicile des patients uniquement, sélectionnez ‘Non applicable’

Non applicable

Oui

Non

**28 - Au cours des 12 derniers mois, à quelle fréquence avez-vous référé des patients aux professionnels de santé suivants** ?

|  | **Jamais** | **Rarement**  (<1/mois) | **Parfois**  (1à 3/mois) | **Régulièrement**  (1 à 2/sem.) | **Très régulièrement**  (>2/sem.) |
| --- | --- | --- | --- | --- | --- |
| Médecin généraliste |  |  |  |  |  |
| Pédiatre |  |  |  |  |  |
| Dentiste |  |  |  |  |  |
| Médecin du sport |  |  |  |  |  |
| Médecin en médecine physique et de réadaptation |  |  |  |  |  |
| Neurologue |  |  |  |  |  |
| Rhumatologue |  |  |  |  |  |
| Gynécologue / gynécologue obstétricien |  |  |  |  |  |
| Médecin du travail |  |  |  |  |  |
| Chirurgien orthopédiste |  |  |  |  |  |
| Neurochirurgien |  |  |  |  |  |
| Kinésithérapeute |  |  |  |  |  |
| Sage-femme / maïeuticien |  |  |  |  |  |
| Podologue |  |  |  |  |  |
| Chiropracteur |  |  |  |  |  |
| Ostéopathe |  |  |  |  |  |
| Autre(s) professionnel(s) de santé (exerçant une profession du champ de la santé réglementée ou non- réglementée) |  |  |  |  |  |

**29 - Au cours des 12 derniers mois, à quelle fréquence les professionnels de santé suivants vous ont-ils référé des patients** ?

|  | **Jamais** | **Rarement**  (<1/mois) | **Parfois**  (1à 3/mois) | **Régulièrement**  (1 à 2/sem.) | **Très régulièrement**  (>2/sem.) |
| --- | --- | --- | --- | --- | --- |
| Médecin généraliste |  |  |  |  |  |
| Pédiatre |  |  |  |  |  |
| Dentiste |  |  |  |  |  |
| Médecin du sport |  |  |  |  |  |
| Médecin en médecine physique et de réadaptation |  |  |  |  |  |
| Neurologue |  |  |  |  |  |
| Rhumatologue |  |  |  |  |  |
| Gynécologue / gynécologue obstétricien |  |  |  |  |  |
| Médecin du travail |  |  |  |  |  |
| Chirurgien orthopédiste |  |  |  |  |  |
| Neurochirurgien |  |  |  |  |  |
| Kinésithérapeute |  |  |  |  |  |
| Sage-femme / maïeuticien |  |  |  |  |  |
| Chiropracteur |  |  |  |  |  |
| Ostéopathe |  |  |  |  |  |
| Autre(s) professionnel(s) de santé (exerçant une profession du champ de la santé réglementée ou non- réglementée) |  |  |  |  |  |

**IV Caractéristiques du ou des lieux d’exercice : localisation, composition et gestion**

**30 - Dans combien de cabinets/structures différentes exercez-vous en tant que chiropracteur(e)** ?

1

2

> 2

Chiropracteur exerçant uniquement au domicile des patients

**31 - Indiquez le nombre d’habitants de la/des communes où se situe(nt) votre/vos lieux d’exercice :**

- Par « commune », il est entendu ville, bourg avec ses villages et hameaux, ou groupe de villages.
- Une à trois réponses sont possibles en fonction du nombre de lieux d’exercice que vous avez précédemment indiqué.
- **Lieu d’exercice n°1** (lieu principal, en termes de temps de travail passé sur site) : … … … … …
- **Lieu d’exercice n°2** (lieu secondaire, en termes de temps de travail passé sur site) : … … … … …
- **Lieu d’exercice n°3**(troisième lieu, en termes de temps de travail passé sur site) : … … … … …

**32 - Indiquez le numéro du ou des département(s) dans lequel ou lesquels vous exercez**:

- Une à trois réponses sont possibles en fonction du nombre de lieux d’exercice que vous avez précédemment indiqué.
- Dans le cas où vous êtes chiropracteur exerçant uniquement au domicile des patients, indiquez le ou les numéros de départements dans le- ou lesquels vous vous déplacez.
- **Lieu d’exercice n°1** (lieu principal, en termes de temps de travail passé sur site) : … … … … …
- **Lieu d’exercice n°2**(lieu secondaire, en termes de temps de travail passé sur site) : … … … … …
- **Lieu d’exercice n°3** (troisième lieu, en termes de temps de travail passé sur site) : … … … … …

**33 - Quelle est votre perception de l’intensité de la concurrence exercée par les autres professionnels de santé proposant des soins de thérapie manuelle** (ex. kinésithérapeutes, médecins, chiropracteurs, ostéopathes) **à proximité de votre principal lieu d’exercice** (c’est à dire celui pour lequel vous consacrez le plus de votre temps de travail) ?

Absence de concurrence

Intensité faible

Intensité moyenne

Forte intensité concurrence

Très forte intensité

**34 - Dispensez-vous régulièrement** (à une fréquence d’au moins une fois par mois) **des soins chiropratiques en dehors de votre ou de vos cabinets**?

Non

Oui, au domicile des patients (sans pour autant que cela constitue mon principal mode d’exercice)

Oui, au sein d’un hôpital public ou privé

Oui, en entreprise

Oui, au sein d’une ou plusieurs association(s) (ex. club sportif)

Oui, dans un ou plusieurs autre(s) contexte(s) que ceux mentionnés ci-dessus (préciser) : … … … … …

**35 - Proposez-vous des consultations dans une ou des langues autres que le français** ?

Oui (précisez) : … … … … …

Non

**36 - Laquelle des situations ci-dessous illustre le mieux votre situation au sein de votre principal lieu d’exercice**?

Dans le cas où vous exercez au domicile des patients uniquement, sélectionnez « Chiropracteur(e) exerçant seul(e) »

Chiropracteur(e) exerçant seul(e)

J’exerce avec au moins un(e) autre chiropracteur(e) et chacun a sa propre salle de consultation.

Je partage ma salle de consultation avec au moins un(e) autre chiropracteur(e).

Chiropracteur(e) en collaboration

Chiropracteur(e) remplaçant(e)

Chiropracteur(e) au sein d’un cabinet/d’une structure pluridisciplinaire (c’est à dire comprenant au minimum deux professionnels de santé aux professions différentes), chacun(e) disposant de sa propre salle de consultation

Autre (préciser) : … … … … …

**37 - Indiquez l’intervalle correspondant le mieux aux honoraires pratiqués pour une première consultation ainsi que celui correspondant le mieux aux honoraires pratiqués pour une consultation de suivi**:

- Ici également, seul votre principal lieu d’exercice, c’est-à-dire celui pour lequel vous consacrez le plus de votre temps de travail hebdomadaire, est à prendre en compte.
- Dans le cas où vous pratiquez des honoraires spéciaux (ex. consultations pédiatriques, partenariats d’entreprises ou associatifs), indiquez uniquement l’intervalle correspondant le mieux aux honoraires les plus régulièrement pratiqués pour une première consultation et pour une consultation de suivi.

|  | **Honoraires pratiqués pour une première consultation** | **Honoraires pratiqués pour une consultation de suivi** |
| --- | --- | --- |
| ≤,30 euros |  |  |
| 31-40 euros |  |  |
| 41-50 euros |  |  |
| 51-60 euros |  |  |
| 61-70 euros |  |  |
| 71-80 euros |  |  |
| 81-90 euros |  |  |
| > 90 euros |  |  |

Non-cross cultural adapted translated version to English (using artificial intelligence)

**Profiles and Activities of Chiropractors in France**

Thank you for agreeing to complete the “*Profiles and Activities of Chiropractors in France*” survey; your contribution is essential!

Depending on your status (e.g., your graduation year, whether or not you have one or more professional activities related to chiropractic outside of clinical practice), you may occasionally be redirected to an additional question or, conversely, to a question whose number does not immediately follow the last question or item you answered. Therefore, please do not pay attention to the numbering of the items and questions, as this is used solely as a reference for data analysis.

If necessary, the survey can be completed in multiple sessions. Your answers are saved each time you move on to the next page of the questionnaire. So, be sure to click through to the next page even if you haven’t finished answering all the questions on the current page, in order not to lose your responses. Likewise, if you expect to be interrupted for 20 minutes or more and thus remain inactive on a page during that time, please save your answers by proceeding to the next page so you can return later without losing any information you’ve already entered.

Lastly, if you were contacted via multiple email addresses, please complete the survey only once and, if you complete it over multiple sessions, always use the same email address.

**I. Individual characteristics**

1. Indicate your age: … … … … …
2. Indicate your gender:

Female
 Male
 Other

1. Indicate the year you obtained your chiropractic degree: … … … … …
2. Indicate the institution from which you obtained your chiropractic degree:

*Institut Franco-Européen de Chiropraxie*
 Anglo-European College of Chiropractic
 Cleveland Chiropractic College
 Life Chiropractic College West
 Life University
 Logan Chiropractic College
 Los Angeles College of Chiropractic
 National University of Health Sciences
 New York Chiropractic College
 Palmer College of Chiropractic
 Palmer College of Chiropractic West
 Sherman Chiropractic College
 Texas Chiropractic College
 Western States Chiropractic College
 Other (specify): …………………

1. If you hold one or more additional degrees beyond your chiropractic qualification, indicate the degree(s) and specialization(s):

- This education may have been completed before or after your chiropractic degree.

Bachelor's degree
Specialization(s): … … … … …

*Diplôme(s) Universitaire(s)* / *Inter-Universitaire (s)*
Specialization(s): … … … … …

Master's degree
Specialization(s): … … … … …

Doctorate
Specialization(s): … … … … …

Other(s) (specify): … … … … …

1. Indicate which French chiropractic-related professional association(s) you are a member of:

None
 Association Française de Chiropraxie
 Syndicat National des Chiropracteurs
 Conseil Français de Chiropraxie du Sport
 Association Française de Chiropraxie Pédiatrique
 Association Française de Chiropraxie Animale
 Association Française pour l’Histoire de la Chiropratique en France
 Other(s) (specify):

1. As a chiropractor, whether or not you are in active clinical practice, indicate the chiropractic-related activity/activities (paid or unpaid) in which you have been involved during the past 12 months:

None
 Teaching (in an academic setting) and/or pedagogical supervision
 Research (as investigator and/or co-author of scientific article(s))
 Administrative/management roles (e.g., at IFEC, AFC, *Fond de Dotation pour la Recherche en Chiropratique*)
 Other(s) (specify): … … … … …

1. Indicate your current status when completing the survey:

I am no longer practicing as a chiropractor but remain engaged in chiropractic-related activities in France.
 I currently practice as a chiropractor (full-time or part-time) in France.

### II. Professional characteristics: weekly working hours and chiropractic-related activities

For the following questions and items, if you work in more than one location, consider your total chiropractic activity.

1. **Indicate the average number of hours per week you devote to chiropractic practice (including patient care and business management):** … … … … …
2. **In your chiropractic practice, how is your weekly working time distributed across the following activities?**

- The total must equal 100%.

|  | 1-25% | 26-50% | 51-75% | 76-100% |
| --- | --- | --- | --- | --- |
| **Patient care** (including history-taking, examination, treatment, counseling, record-keeping, interprofessional communication) | □ | □ | □ | □ |
| **Business management** (e.g., administration, accounting, staff management if applicable, communication) | □ | □ | □ | □ |
|  |  |  |  |  |

1. **On average, how much time do you spend with a new patient (initial consultation)?**

≤ 5 min

6–10 min

11–15 min

16–20 min

21–30 min

31–45 min

46–60 min

> 60 min

**12. On average, how much time do you spend with a patient during a follow-up visit?**

≤ 5 min

6–10 min

11–15 min

16–20 min

21–30 min

31–45 min

46–60 min

> 60 min

**13. On average, how many consultations do you personally conduct per week?**

≤ 10

11–20

21–30

31–40

41–50

51–60

61–70

71–80

81–90

91–100

101–110

> 111

**14. Indicate the average number of new patients you personally see per week:**

0

1–3

4–6

7–9

10–12

13–15

> 15

**15. On average, within what timeframe do you see a patient presenting with acute or subacute symptoms (lasting less than 3 months)?**

Same day

1–2 days

3–4 days

5–7 days

1–2 week

> 2 weeks

**16. On average, within what timeframe do you see a patient presenting with chronic symptoms (lasting more than 3 months)?**

Same day

1–2 days

3–4 days

5–7 days

1–2 week

> 2 weeks

**17. If you have various paid chiropractic-related activities, is your clinical practice your main activity in terms of weekly working hours (not earnings)?**

Not applicable (if clinical practice takes up 100% of your weekly work time)

Yes

No

**18. Indicate the type(s) of continuing education activities you participated in over the past 12 months:**

Not applicable (if you graduated less than 12 months ago)

I did not participate in any continuing education activity in the past 12 months.

University degree program (Bachelor’s, DU/DIU, Master’s, PhD)

Seminar(s) on chiropractic topics or related to chiropractic practice (in-person and/or online)

Scientific and/or science communication conference(s) (in-person and/or online)

Online course (MOOC or equivalent)

Reading of scientific articles and/or academic works (e.g., clinical practice guidelines, doctoral theses)

Other(s) (e.g., participation in research projects) (specify): … … … … …

**19. Indicate the average number of hours you devoted to continuing education activities over the past 12 months:**

1–10 hours

11–20 hours

21–30 hours

31–40 hours

41–50 hours

51–60 hours

61–70 hours

71–80 hours

81–90 hours

91–100 hours

> 100 hours

### II. Clinical practice: patients, diagnostic Imaging, therapeutic modalities/techniques, and interprofesionnal referrals

**20. On average each week and based on your overall practice (i.e., including all your work locations), what proportion of patients consult you for the following reasons (as their main reason for consultation)?**

- If you do not treat any patients for a particular reason, check "None".
- The total proportions must equal 100%.

|  | **None** | **1-10%** | **11-20%** | **21-30%** | **31-40%** | **41-50%** | **51-60%** | **61-70**  **%** | **71-80**  **%** | **81-90%** | **91-100%** |
| --- | --- | --- | --- | --- | --- | --- | --- | --- | --- | --- | --- |
| Headaches (tension-type and/or cervicogenic) |  |  |  |  |  |  |  |  |  |  |  |
| Low back/pelvis pain without leg pain |  |  |  |  |  |  |  |  |  |  |  |
| Low back/pelvis pain with leg pain |  |  |  |  |  |  |  |  |  |  |  |
| Mid-back pain with or without irradiation |  |  |  |  |  |  |  |  |  |  |  |
| Neck pain without arm pain |  |  |  |  |  |  |  |  |  |  |  |
| Neck pain with arm pain |  |  |  |  |  |  |  |  |  |  |  |
| Lower extremity disorder |  |  |  |  |  |  |  |  |  |  |  |
| Upper extremity disorder |  |  |  |  |  |  |  |  |  |  |  |
| Temporomandibular joint dysfunction syndrome |  |  |  |  |  |  |  |  |  |  |  |
| Prevention (secondary or tertiary of a musculoskeletal disorder) |  |  |  |  |  |  |  |  |  |  |  |
| Other musculoskeletal disorder (i.e., not listed above) |  |  |  |  |  |  |  |  |  |  |  |
| Non-musculoskeletal disorder (e.g., migraine) |  |  |  |  |  |  |  |  |  |  |  |

**21. Based on your overall practice, indicate the proportion of patients you treat in each of the following age categories:**

- If you do not treat any patients in one or more categories, select "None".
- The total proportions must equal 100%.

|  | **None** | **1-10%** | **11-20%** | **21-30%** | **31-40%** | **41-50%** | **51-60%** | **61-70%** | **71-80%** | **81-90%** | **91-100%** |
| --- | --- | --- | --- | --- | --- | --- | --- | --- | --- | --- | --- |
| **< 6 mth** |  |  |  |  |  |  |  |  |  |  |  |
| **6 mth - 5 yrs** |  |  |  |  |  |  |  |  |  |  |  |
| **6 - 14 yrs** |  |  |  |  |  |  |  |  |  |  |  |
| **15 – 24 yrs** |  |  |  |  |  |  |  |  |  |  |  |
| **25 – 39 yrs** |  |  |  |  |  |  |  |  |  |  |  |
| **40 - 64 yrs** |  |  |  |  |  |  |  |  |  |  |  |
| **≥ 65 yrs** |  |  |  |  |  |  |  |  |  |  |  |

**22. Which of the following best describes your usual approach when X-rays are indicated?**

I most often request X-rays directly from a radiologist.

I most often ask the patient to request them from their general practitioner (without a referral letter).

I most often ask the patient to request them from their general practitioner (with a referral letter).

**23. Which of the following best describes your usual approach when ultrasound is indicated?**

|  |  | **I perform myself the diagnosis imaging** | **I refer to radiologist** | **I refer to patient’s general practitioner without referral letter** | **I refer to patient’s general practitioner with referral letter** |
| --- | --- | --- | --- | --- | --- |
| **Musculoskeletal ultrasound** |  |  |  |  |  |
| **Non-musculoskeletal ultrasound** |  | Not applicable |  |  |  |

**24. Which of the following best describes your usual approach when a CT scan or MRI is indicated?**

I most often request the CT scan or MRI directly from a radiologist.

I most often ask the patient to request it from their general practitioner (without a referral letter).

I most often ask the patient to request it from their general practitioner (with a referral letter).

**25. For each of the following therapeutic modalities or techniques, indicate the percentage range that best represents the proportion of patients you apply it to:**

- If you do not use a particular modality or technique, leave the line blank.

|  | **1-25%** | **26-50%** | **51-75%** | **76-100%** |
| --- | --- | --- | --- | --- |
| High velocity and low amplitude manipulation* (including *Diversified* and *Gonstead* techniques) |  |  |  |  |
| Instrumentally assisted manipulation* (including *Activator Methods*) |  |  |  |  |
| Mechanically assisted manipulation* (including *Thompson* and *Hole In One techniques*) |  |  |  |  |
| Spinal traction (including *Cox* technique) |  |  |  |  |
| Vertebral mobilization (e.g., *Mc Kenzie*, *Mulligan* and *Maitland* techniques) |  |  |  |  |
| Orthopedic blocking |  |  |  |  |
| Passive myofascial techniques (e.g., stretching, massage, *Trigger Point*, *Graston* and *Dry Needling* techniques) |  |  |  |  |
| Physical therapy modalities (e.g., transcutaneous electric nerve stimulation, cryotherapy) |  |  |  |  |
| Taping (e.g., *Kinesio taping* technique) |  |  |  |  |
| Strapping and/or advices related to orthotic devices (e.g., bracing) |  |  |  |  |
| Exercises directed to chief complaint (supervised or not, e.g., stretching, strengthening) |  |  |  |  |
| *Applied kinesiology* |  |  |  |  |
| *Bio energetic synchronization technique* |  |  |  |  |
| *Network technique* (n=387) |  |  |  |  |
| *Sacrooccipital technique* |  |  |  |  |
| Information specific to the chief complaint (e.g., its natural course, reinsurance) |  |  |  |  |
| Counseling on daily living habits (e.g., physical activity, nutrition, sleep) |  |  |  |  |
| Other(s) |  |  |  |  |

**Modality that can be delivered at the spine and/or extremities*.

**26. Do you provide your patients with rehabilitation equipment (e.g., resistance bands, massage balls/rollers)?**

Yes

No

**27. Do you have a room dedicated to rehabilitation at one or more of your practice locations?**

- If you only practice at patients’ homes, select “Not applicable”.

Not applicable

Yes

No

**28. Over the past 12 months, how often have you referred patients to the following healthcare professionals?**

|  | **Never** | **Rarely**  (<1/mth) | **Sometimes**  (1-3/mth) | **Often**  (1-2/wk) | **Routinely**  (>2/wk) |
| --- | --- | --- | --- | --- | --- |
| General practitioner |  |  |  |  |  |
| Pediatrician |  |  |  |  |  |
| Dentist |  |  |  |  |  |
| Sports doctor |  |  |  |  |  |
| Doctor specialized in physical and rehabilitation medicine |  |  |  |  |  |
| Neurologist |  |  |  |  |  |
| Rheumatologist |  |  |  |  |  |
| Gynecologist ± obstetrician |  |  |  |  |  |
| Occupational physician |  |  |  |  |  |
| Orthopedic surgeon |  |  |  |  |  |
| Neurosurgeon |  |  |  |  |  |
| Physiotherapist |  |  |  |  |  |
| Midwife/maieutician |  |  |  |  |  |
| Podiatrist |  |  |  |  |  |
| Chiropractor |  |  |  |  |  |
| Osteopath |  |  |  |  |  |
| Other(s) (whether regulated or not) |  |  |  |  |  |

**29. Over the past 12 months, how often have the following healthcare professionals referred patients to you?**

|  | **Never** | **Rarely**  (<1/mth) | **Sometimes**  (1-3/mth) | **Often**  (1-2/wk) | **Routinely**  (>2/wk) |
| --- | --- | --- | --- | --- | --- |
| General practitioner |  |  |  |  |  |
| Pediatrician |  |  |  |  |  |
| Dentist |  |  |  |  |  |
| Sports doctor |  |  |  |  |  |
| Doctor specialized in physical and rehabilitation medicine |  |  |  |  |  |
| Neurologist |  |  |  |  |  |
| Rheumatologist |  |  |  |  |  |
| Gynecologist ± obstetrician |  |  |  |  |  |
| Occupational physician |  |  |  |  |  |
| Orthopedic surgeon |  |  |  |  |  |
| Neurosurgeon |  |  |  |  |  |
| Physiotherapist |  |  |  |  |  |
| Midwife/maieutician |  |  |  |  |  |
| Chiropractor |  |  |  |  |  |
| Osteopath |  |  |  |  |  |
| Other(s) (whether regulated or not) |  |  |  |  |  |

### IV. Practice characteristics: location, composition, and management

**30. In how many different clinics/facilities do you practice as a chiropractor?**

1

2

> 2

Chiropractor working exclusively at patients’ homes

**31. Indicate the population size of the town(s)/municipality(ies) where your practice location(s) are situated:**

- Up to three answers possible depending on how many practice locations you previously indicated.
- Practice location 1 (primary location based on time spent): ................
- Practice location 2 (secondary location): ................
- Practice location 3 (third location): … … … … …

**32. Indicate the department number(s) in which you practice:**

- Up to three answers possible depending on the number of practice locations previously indicated.
- If you are a chiropractor working exclusively at patients’ homes, indicate the department number(s) you cover.
- Practice location 1: … … … … …
- Practice location 2: … … … … …
- Practice location 3: … … … … …

**33. What is your perception of the intensity of competition from other healthcare professionals providing manual therapy (e.g., physical therapists, physicians, chiropractors, osteopaths) near your main practice location (i.e., where you spend most of your work time)?**

No competition

Low

Moderate

Intense

Very intense

**34. Do you regularly (at least once a month) provide chiropractic care outside your clinic(s)?**

No

At patients’ homes (without this being my main mode of practice)

In a public or private hospital

In industry / industries

In non**-**profit organization(s) (e.g., sports club)

In one or more other contexts (specify): … … … … …

**35. Do you offer consultations in any language(s) other than French?**

Yes (specify): … … … … …

No

**36. Which of the following best describes your situation within your main practice location?**

- If you practice only at patients’ homes, select “Solo practitioner”.

Solo practitioner

I work with at least one other chiropractor, each with their own treatment room.

I share my treatment room with at least one other chiropractor.

Associate chiropractor

Replacement chiropractor

Chiropractor in a multidisciplinary clinic/facility (i.e., including at least two different healthcare professions), each with their own treatment room

Other (specify): … … … … …

**37. Indicate the fee range that best corresponds to the rate you charge for an initial consultation and for a follow-up visit:**

- Only your main practice location (i.e., where you spend most of your weekly working time) should be considered.

|  | **Initial**  **consultation** | **Follow-up consultation** |
| --- | --- | --- |
| ≤ €30 | □ | □ |
| €31–40 | □ | □ |
| €41–50 | □ | □ |
| €51–60 | □ | □ |
| €61–70 | □ | □ |
| €71–80 | □ | □ |
| €81–90 | □ | □ |
| > €90 | □ | □ |
