# Supplementary material for: Contemporary profiles and professional activities of French chiropractors: a national survey
Source: Chiropr Man Therap. 2025 Oct 13;33:43. doi: 10.1186/s12998-025-00602-2 (PMC12516846; doi:10.1186/s12998-025-00602-2)
Supplement: Supplementary file 2 — Supplementary Material 2 [file 12998_2025_602_MOESM2_ESM.docx]

**Additional file 2**: Supplementary tables of results (1A to 13A)

**Table 1A**. Additional participants’ characteristics

| ***Gender*** (n=495) | % (n) |
| --- | --- |
| Female | 67.7% (n=335) |
| Male | 31.9% (n=158) |
| Other | 0.4% (n=2) |
| ***Place of graduation*** (n=494) | % (n) |
| Anglo-European College of Chiropractic | 0.4% (n=2) |
| Cleveland Chiropractic College | 1% (n=5) |
| *Institut Franco-Européen de Chiropraxie* | 94.5% (n=467) |
| Life Chiropractic College West | 1.4% (n=7) |
| Life University | 0.2% (n=1) |
| National University of Health Sciences | 0.4% (n=2) |
| Palmer College of Chiropractic | 0.6% (n=3) |
| Palmer College of Chiropractic West | 0.4% (n=2) |
| Parker University | 0.2% (n=1) |
| Sherman Chiropractic College | 0.6% (n=3) |
| *Université du Québec à Trois Rivières* | 0.2% (n=1) |
| ***Professional association membership(s)**** (n=494) | % (n) |
| *Association Française de Chiropraxie* | 92.3% (n=456) |
| *Association Française de Chiropraxie Animale* | 4.3% (n=21) |
| *Association Française de Chiropraxie Pédiatrique* | 28.1 (n=139) |
| *Association Française pour l’Histoire de la Chiropraxie en France* | 1.6% (n=8) |
| *Conseil Français de Chiropraxie du Sport* | 6.1% (n=30) |
| *Syndicat National des Chiropracteurs* | 0.8% (n=4) |
| Other (mainly members of the French branch of the *International College of Applied Kinesiology* n=19/34) | 6.9% (n=34) |
| None | 4.5% (n=22) |

**Respondents could indicate being member of one or several French chiropractic associations.*

**Table 2A**. Working hours per week (n=458)

| ***Total working hours*** / ***week*** | % (n) |
| --- | --- |
| < 7hr | 2.6% (n=12) |
| ≥ 7hr to < 14hr | 3.5% (n=16) |
| ≥ 14hr to < 21hr | 13.1% (n=60) |
| ≥ 21hr to < 28hr | 10.7% (n=49) |
| ≥ 28hr to < 35hr | 20.3% (n=93) |
| ≥ 35hr to 39hr | 14.8% (n=68) |
| > 39hr | 34.9% (n=160) |

**Table 3A**. Time devoted to patient’s care and administrative duties per week

| ***Patient care*** (n=471) | % (n) | ***Administrative duties*** (n=469) | % (n) |
| --- | --- | --- | --- |
| 1-25% | 0.6% (n=3) | 1-25% | 88.5% (n=415) |
| 26-50% | 2.1% (n=10) | 26-50% | 10.2% (n=48) |
| 51-75% | 36.5% (n=172) | 51-75% | 0.85% (n=4) |
| 76-100% | 60.7% (n=286) | 76-100% | 0.4% (n=2) |

**Table 4A**. Mean number of patients seen per week

| ***Total nb patients*/*week*** (n=462) | % (n) | ***New patients*/*week*** (n=464) | % (n) |
| --- | --- | --- | --- |
| ≤ 10 | 5.6% (n=26) | 0 | 0.4% (n=2) |
| 11-20 | 15.4% (n=71) | 1-3 | 13.4% (n=62) |
| 21-30 | 19.7% (n=91) | 4-6 | 36.6% (n=170) |
| 31-40 | 21.4% (n=99) | 7-9 | 31.5% (n=146) |
| 41-50 | 17.75% (n=82) | 10-12 | 12.3% (n=57) |
| 51-60 | 8.7% (n=40) | 13-15 | 2.2% (n=10) |
| 61-70 | 4.55% (n=21) | > 15 | 3.7% (n=17) |
| 71-80 | 3% (n=14) |  |  |
| 81-90 | 1.5% (n=7) |  |  |
| 91-100 | 0.9% (n=4) |  |  |
| 101-110 | 0.4% (n=2) |  |  |
| > 110 | 1.1% (n=5) |  |  |

**Table 5A**. Patient wait time before getting an appointment for a chief complaint

| ***Acute***/***subacute chief complaint*** (n=462) | % (n) | ***Chronic chief complaint*** (n=464) | % (n) |
| --- | --- | --- | --- |
| Same day | 14.1% (n=65) | Same day | 7.1% (n=33) |
| 1-2 days | 47.2% (n=218) | 1-2 days | 24.1% (n=112) |
| 3-4 days | 21.2% (n=98) | 3-4 days | 30.2% (n=140) |
| 5-7 days | 8.9% (n=41) | 5-7 days | 16.8% (n=78) |
| 1-2 week(s) | 6.5% (n=30) | 1-2 week(s) | 15.5% (n=72) |
| > 2 weeks | 2.2% (n=10) | > 2 weeks | 6.25% (n=29) |

**Table 6A**. Average time spent with new patient and for subsequent appointments

| ***New patient*** (n=472) | % (n) | ***Subsequent appointment*** (n=471) | % (n) |
| --- | --- | --- | --- |
| 6-10 min | 0 | 6-10 min | 1.1% (n=5) |
| 11-15 min | 0 | 11-15 min | 2.5% (n=12) |
| 16-20 min | 1.3% (n=6) | 16-20 min | 8.3% (n=39) |
| 21-25 min | 0.6% (n=3) | 21-25 min | 9.1% (n=43) |
| 26-30 min | 5.9% (n=28) | 26-30 min | 43.7% (n=206) |
| 31-35 min | 7.2% (n=34) | 31-35 min | 14% (n=66) |
| 36-40 min | 10% (n=47) | 36-40 min | 8.3% (n=39) |
| 41-45 min | 25.6% (n=121) | 41-45 min | 9.8% (n=46) |
| 46-50 min | 13.8% (n=65) | 46-50 min | 1.9% (n=9) |
| 51-55 min | 3.4% (n=16) | 51-55 min | 0.2% (n=1) |
| 56-60 min | 27.1% (n=128) | 56-60 min | 0.8% (n=4) |
| > 60 min | 5.1% (n=24) | > 60 min | 0.2% (n=1) |

**Table 7A**. Time spent in continuing education activities in the past twelve months (n=370*)

| ***Number of hours of continuing education*** | % (n) |
| --- | --- |
| 1-10 h | 8.9% (n=33) |
| 11-20 h | 12.2% (n=45) |
| 21-30 h | 15.4% (n=57) |
| 31-40 h | 13.2% (n=49) |
| 41-50 h | 13% (n=48) |
| 51-60 h | 8.1% (n=30) |
| 61-70 h | 3% (n=11) |
| 71-80 h | 4.9% (n=18) |
| 81-90 h | 2.7% (n=10) |
| 91-100 h | 3.5% (n=13) |
| > 100 h | 15.1% (n=56) |

**Participants were concerned by this item only if they reported having been engaged in at least one continuing education activity in the past twelve months*.

**Table 8A**. Additional practice characteristics

| ***Number of practice locations*** (n=378) | % (n) |
| --- | --- |
| One | 74.9% (n=283) |
| Two | 22.5% (n=85) |
| More than two | 2.7% (n=10) |
| Practicing exclusively in patients’ home | None |
| ***Mono-***/***multidisciplinary setting*** (n=372) | % (n) |
| Monodisciplinary | 72.6% (n=270) |
| *Solo practice* | 42.2% (n=114) |
| *Work with one or several other chiropractors* | 54.1% (n=146) |
| *Replacement chiropractor* | 3.7% (n=10) |
| Multidisciplinary | 27.4% (n=102) |
| ***Care delivery outside office*** (n=378) | % (n) |
| Yes | 17.5% (n=66) |
| *Patient houses* | 56% (n=37) |
| *Industries* | 21.2% (n=14) |
| *Non-for-profit organizations* | 15.15% (n=10) |
| *Public or private hospitals* | 1.5% (n=1) |
| *Others contexts (e.g., retirement homes)* | 18.2% (n=12) |
| No | 82.5% (n=312) |
| ***Consultation in one or several languages other than French*** (n=379) | % (n) |
| Yes* | 63.1% (n=239) |
| *English* | 97.5% (n=233) |
| *Spanish* | 15.9% (n=38) |
| *Italian* | 5% (n=12) |
| No | 36.9% (n=140) |

**Only the three most mentioned languages are reported in the table*.

**Table 9A**. Practice geographical location in France (main, 2^nd^ and 3^rd^ practices) and number of inhabitants per practice location

| ***Region of the main practice*** (n=368) | % (n) |
| --- | --- |
| *Ile-de-France* | 20.1% (n=74) |
| *Auvergne-Rhône-Alpes* / *Occitanie* | 12% (n=44, each region) |
| *Nouvelle-Aquitaine* | 9.5% (n=35) |
| *Grand-Est* | 7.9% (n=29) |
| *Bretagne* | 6.8% (n=25) |
| *Normandie* / *Provence-Alpes-Côte d’Azur* | 6.25% (n=23, each region) |
| *Bourgogne-Franche-Comté* | 5.4% (n=20) |
| *Pays de la Loire* | 4.9% (n=18) |
| *Hauts-De-France* | 4.6% (n=17*) |
| *Centre-Val de Loire* | 3.5% (n=13) |
| *Régions ultramarines* or *Collectivités d’outre-mer* | 0.8% (n=3) |
| ***Region of 2^nd^ place of practice*** (n=88) | % (n) |
| *Ile-de-France* | 13.6% (n=12) |
| *Nouvelle-Aquitaine* | 12.5% (n=11) |
| *Auvergne-Rhône-Alpes* / *Bourgogne-Franche-Comté* / *Normandie* / *Occitanie* | 9% (n=8, each region) |
| *Pays de la Loire*/ *Grand-Est* | 7.95% (n=7*, each region) |
| *Bretagne* / *Provence-Alpes-Côte d’Azur* | 6.8% (n=6, each region) |
| *Centre-Val de Loire* / *Hauts-De-France* | 3.4% (n=3, each region) |
| *Régions ultramarines ou collectivité d’outre-mer* | 1.1% (n=1) |
| ***Region of the 3^rd^ place of practice*** (n=9) | % (n) |
| *Bourgogne-Franche-Comté* / *Normandie* / *Occitanie* | 22.2% (n=2, each region) |
| *Auvergne-Rhône-Alpes* / *Grand-Est* / *Ile-de-France* | 11.1% (n=1, each region) |
| ***Population of the main practice location*** (n=375) | % (n) |
| ≥ 2 000 000 | 6.1% (n=23) |
| 200 000 - 1 999 990 | 10.7% (n=40) |
| 100 000 - 199 999 | 10.1% (n=38) |
| 50 000 - 99 999 | 12.8% (n=48) |
| 20 000 - 49 999 | 21.1% (n=79) |
| 10 000 - 19 999 | 18.1% (n=68) |
| 5000 -9999 | 11.5% (n=43) |
| 2000 - 4999 | 6.9% (n=26) |
| < 2000 | 2.7% (n=10) |
| ***Population of the 2^nd^ practice location*** (n=92) | % (n) |
| ≥ 2 000 000 | 2.2% (n=2) |
| 200 000 - 1 999 990 | 6.5% (n=6) |
| 100 000 - 199 999 | 6.5% (n=6) |
| 50 000 - 99 999 | 9.8% (n=9) |
| 20 000 - 49 999 | 22.8% (n=21) |
| 10 000 - 19 999 | 27.2% (n=25) |
| 5000 -9999 | 10.9% (n=10) |
| 2000 - 4999 | 7.6% (n=7) |
| < 2000 | 6.5% (n=6) |
| ***Population of the 3^rd^ practice location*** (n=10) | % (n) |
| 50 000 - 99 999 | 10% (n=1) |
| 20 000 - 49 999 | 30% (n=3) |
| 10 000 - 19 999 | 30% (n=3) |
| < 2000 | 30% (n=3) |

**Including one chiropractor having its 2^nd^ place of practice abroad*.

***Including (for Grand-Est) one chiropractor having its 1^st^ place of practice abroad*.

**Table 10A**. Service charges for initial and subsequent care (n=377)

| ***New patient*** | % (n) | ***Subsequent visits*** | % (n) |
| --- | --- | --- | --- |
| < 30 eu | 0.5% (n=2) | < 30 eu | 0 |
| 31-40 eu | 0.8% (n=3) | 31-40 eu | 4% (n=15) |
| 41-50 eu | 25.2% (n=95) | 41-50 eu | 41.6% (n=157) |
| 51-60 eu | 46.15% (n=174) | 51-60 eu | 41.6% (n=157) |
| 61-70 eu | 19.4% (n=73) | 61-70 eu | 10.3% (n=39) |
| 71-80 eu | 4.5% (n=17) | 71-80 eu | 1.85% (n=7) |
| 81-90 eu | 2.4% (n=9) | 81-90 eu | 0 |
| > 90 eu | 1.1% (n=4) | > 90 eu | 0.5% (n=2) |

**Table 11A**. Perceived level of competition with other manual therapy providers near the main practice (n=376)

| ***Perceived intensity*** | **None** | **Low** | **Medium** | **Intense** | **Very intense** |
| --- | --- | --- | --- | --- | --- |
| % (n) | 5.05%  (n=19) | 32.7%  (n=123) | 30.6%  (n=115) | 22.3%  (n=84) | 9.3%  (n=35) |

**Table 12A**. Exercise facilities, including movable equipment, offered in the chiropractic clinics

| ***Movable equipment*** (n=379) | % (n) | ***Room exercise at the clinic*** (n=361) | % (n) |
| --- | --- | --- | --- |
| Yes | 27.2% (n=103) | Yes | 1.9% (n=7) |
| No | 72.8% (n=276) | No | 98.1% (n=354) |

**Table 13A**. How chiropractors refer when a diagnosis imaging is needed (musculoskeletal ultrasound, non-musculoskeletal ultrasound, X-rays, and CT-scan or MRI)?

| **Way of referring** | **Musculoskeletal ultrasound** (n=384) | **Non-musculoskeletal ultrasound** (n=372) | **X-rays** (n=387) | **CT-scan or MRI** (n=387) |
| --- | --- | --- | --- | --- |
| Direct referral to radiologist | 8.6% (n=33) | 3.5% (n=13) | 11.4% (n=44) | 5.7% (n=22) |
| Referral to patient’s general practitioner with referral letter | 21.6% (n=83) | 24.7% (n=92) | 22.7% (n=88) | 36.2% (n=140) |
| Referral to patient’s general practitioner without referral letter | 68.2% (n=262) | 71.8% (n=267) | 65.9% (n=255) | 58.1% (n=225) |
| Perform themselves the diagnosis imaging | 1.6% (n=6) | NA* | NA* | NA* |

**In France, only medical doctors are allowed to perform this diagnosis imaging*.
