# Supplementary material for: Contemporary profiles and professional activities of French chiropractors: a national survey
Source: Chiropr Man Therap. 2025 Oct 13;33:43. doi: 10.1186/s12998-025-00602-2 (PMC12516846; doi:10.1186/s12998-025-00602-2)
Supplement: Supplementary file 3 — Supplementary Material 3 [file 12998_2025_602_MOESM3_ESM.docx]

**Additional file 3**: Supplementary table of results (Table 1B)

**Table 1B**. Percentages of patients within specific age groups seeking chiropractic care

| Age group | None | 1-10% | 11-20% | 21-30% | 31-40% | 41-50% | 51-60% | 61-100% |
| --- | --- | --- | --- | --- | --- | --- | --- | --- |
| < 6 months  (n=370) | 28.65%  (n=106) | 50%  (n=185) | 13.5%  (n=50) | 5.7%  (n=21) | 1.1%  (n=4) | 0 | 0.5%  (n=2) | 0.5%  (n=2) |
| 6 months - 5 yrs  (n=372) | 22%  (n=82) | 62.6%  (n=233) | 11.3%  (n=42) | 3 .5%  (n=13) | 0 | 0.3%  (n=1) | 0 | 0.3%  (n=1) |
| 6-14 yrs  (n=373) | 6.2% (n=23) | 78.8% (n=294) | 12.3% (n=46) | 1.9% (n=7) | 0.5% (n=2) | 0.3%  (n=1) | 0 | 0 |
| 15-24 yrs  (n=375) | 1.9%  (n=7) | 39.5%  (n=148) | 43.2%  (n=162) | 11.2%  (n=42) | 2.7%  (n=10) | 1.1%  (n=4) | 0.3%  (n=1) | 0.3%  (n=1) |
| **25-39 yrs**  (n=379) | 0 | 4.2%  (n=16) | **28.2%**  (n=107) | **35.1%**  (n=133) | **17.9%**  (n=68) | 8.2%  (n=31) | 2.9%  (n=11) | 3.4%  (n=13) |
| **40-64 yrs**  (n=382) | 0 | 2.6%  (n=10) | **16.5%**  (n=63) | **29.1%**  (n=111) | **28.5%**  (n=109) | 13.1%  (n=50) | 5.2%  (n=20) | 5%  (n=19) |
| ≥ 65 yrs  (n=379) | 0.8%  (n=3) | 21.6%  (n=82) | 30.1%  (n=114) | 27.7%  (n=105) | 11.6%  (n=44) | 2.9%  (n=11) | 2.6%  (n=10) | 2.6%  (n=10) |

*The three most frequent categories of the two most often mentioned age groups are in* ***bold****.*
